# Characteristics of First Nations patients who take their own leave from an inner‐city emergency department, 2016–2020

**DOI:** 10.1111/1742-6723.14057

**Published:** 2022-08-30

**Authors:** Jennie Hutton, Tilini Gunatillake, Deborah Barnes, Georgina Phillips, Jacqueline Maplesden, Andrew Chan, Prudence Shanahan, Rachel Zordan, Vijaya Sundararajan, Kerry Arabena, Alyssa Quigley, T'ia Pynor‐Greedy, Toni Mason

**Affiliations:** ^1^ Emergency Department St Vincent's Hospital Melbourne Melbourne Victoria Australia; ^2^ Department of Medicine Faculty of Medicine, Dentistry and Health Sciences, The University of Melbourne Melbourne Victoria Australia; ^3^ Victorian Comprehensive Cancer Centre The University of Melbourne Melbourne Victoria Australia; ^4^ Aboriginal Health Unit St Vincent's Hospital Melbourne Melbourne Victoria Australia; ^5^ School of Public Health and Preventive Medicine Monash University Melbourne Victoria Australia; ^6^ Complex Care Services St Vincent's Hospital Melbourne Melbourne Victoria Australia; ^7^ Department of Psychiatry St Vincent's Hospital Melbourne Melbourne Victoria Australia; ^8^ Education and Learning St Vincent's Hospital Melbourne Melbourne Victoria Australia; ^9^ Karabena Consulting Melbourne Victoria Australia; ^10^ Melbourne School of Population and Global Health The University of Melbourne Melbourne Victoria Australia; ^11^ St Vincent's Hospital Melbourne Melbourne Victoria Australia

**Keywords:** did not wait, emergency department, indigenous, left without being seen

## Abstract

**Objective:**

Using a strength‐based framework, we aimed to describe and compare First Nations patients who completed care in an ED to those who took their own leave.

**Methods:**

Routinely collected adult patient data from a metropolitan ED collected over a 5‐year period were analysed.

**Results:**

A total of 6446 presentations of First Nations patients occurred from 2016 to 2020, constituting 3% of ED presentations. Of these, 5589 (87%) patients waited to be seen and 857 (13%) took their own leave. Among patients who took their own leave, 624 (73%) left not seen and 233 (27%) left at own risk after starting treatment. Patients who were assigned a triage category of 4–5 were significantly more likely to take their own leave (adjusted odds ratio [OR] 3.17, 95% confidence interval [CI] 2.67–3.77, *P* < 0.001). Patients were significantly less likely to take their own leave if they were >60 years (adjusted OR 0.69, 95% CI 1.01–1.36, *P* = 0.014) and had private health insurance (adjusted OR 0.61, 95% CI 0.45–0.84, *P* < 0.001). Patients were more likely to leave if they were women (adjusted OR 1.17, 95% CI 1.01–1.36, *P* = 0.04), had an unknown housing status (adjusted OR 1.76, 95% CI 1.44–2.15, *P* < 0.001), were homeless (adjusted OR 1.50, 95% CI 1.22–1.93, *P* < 0.001) or had a safety alert (adjusted OR 1.60, 95% CI 1.35–1.90, *P* < 0.001).

**Conclusion:**

A lower triage category is a strong predictor of First Nations patients taking their own leave. It has been documented that First Nations patients are under‐triaged. One proposed intervention in the metropolitan setting is to introduce practices which expediate the care of First Nations patients. Further qualitative studies with First Nations patients should be undertaken to determine successful approaches to create equitable access to emergency healthcare for this population.

## Graphical abstract

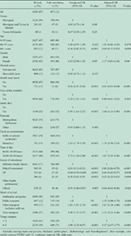

A comparison of First Nations patients in an inner‐city ED who waited to those who left the ED not seen had a higher triage category. In this setting, a higher triage category could be explored as a way of improving health equity for First Nations patients.

**Key findings**

- First Nations patients are more likely to stay to complete their ED treatment if they are triaged at a higher category and seen within the first hour of presentation.
- A social determinants of health lens and trauma informed care are recommended for best practice in ED care for First Nations patients.

## Introduction

Strategies determined by First Nations people to enhance cultural safety and cultural responsiveness of health institutions are necessary to accomplishing a ‘racism free’ health service delivery system, capable of addressing health inequalities between Australia's First People and the general population.^1^ Patients who take their own leave (TOL) from the ED, leave the hospital either before they have a consultation with an ED clinician, termed ‘left not seen’ (LNS), or before treatment is completed, termed ‘left at own risk’ (LOR). The number of First Nations patients who TOL from hospital is increasing,^2^ and these patients are evidently not having their needs met. In Australian metropolitan EDs the LNS rate for First Nations patients is 9.9%, compared to 5.6% for non‐First Nations patients.^3^ We have chosen to use the terms ‘left not seen’ as opposed to the corresponding term ‘did not wait’. Similarly, ‘left at own risk’ is preferred to the equivalent ‘discharge (or left) against medical advice’. This is in addition to using ‘take own leave’ as an overarching term to describe both events. These terms are chosen so as not to stigmatise patients whose reason for leaving may not be understood.

Previous studies indicate that patients of colour are ‘under‐triaged’ when compared with other patients, and consequently have longer wait times for the same condition.^4^, ^5^ The difference in these rates could be attributable to unconscious bias, racism and discriminatory practices in EDs, and are relevant for the design, delivery and evaluation of ED processes. The “Flexiclinic” approach at St Vincent's Hospital, Sydney, is an example of an innovative approach to cultural safety which has demonstrated a reduction in the number of First Nations patients who TOL.^6^, ^7^ Importantly, this approach identified strengths and resilience of Indigenous peoples in overcoming challenges despite the adversity and hardship with which they are confronted.^8^ There is currently little known about protective factors that support First Nations patients to wait for care, and how these are linked to social and cultural determinants of health and well‐being.^9^

Our paper aims to describe the characteristics of First Nations patients who TOL (either LNS or LOR) compared to other First Nations patients who have completed care in the ED. The aim is to promote discussion about alternative ED‐based practices that promote resilience and addresses institutional racism.

## Methods

### Study design and setting

The investigation was a retrospective study of adult First Nations patients who presented to ED over a 5‐year period using descriptive outcomes. The setting is an inner‐city tertiary adult hospital in Melbourne, Australia, between January 2016 and December 2020. The hospital has 504 inpatient beds, and the ED provides approximately 46 000 episodes of care annually to individuals residing in state correctional facilities.

### Participants

Participants were included if they identified upon registration at the ED as Aboriginal and/or Torres Strait Islander, were 16 years or older and presented during the study period. To counteract the deficit framing that commonly pervades research we used a strength‐based method to identify factors that are associated with First Nations patients who wait to be seen.^10^ First Nations patients who TOL (either LNS or LOR) were compared with those who completed care.

### Variables

Variables of interest (patient characteristics and variables specific to the ED episode) were obtained from data routinely collected in the patient electronic medical record (Table 1). Records with missing data were excluded from the analysis. Patients were considered in ‘stable accommodation’ if they lived in a private residential property, resided in correctional facilities or nursing homes. Patients were considered homeless if they were unsheltered (residing in public space), living in homeless shelters, supported accommodation, shelters, refuges or boarding hostels.^11^ Other variables are detailed in Table 1. Safety alerts are applied if staff have perceived threatening behaviour by the patient previously, and subsequently requested an alert applied to the patient's records. ‘Time waited’ variables were not analysed as the exact time of departure was not accurately recorded. For ‘ED busyness’ markers, ‘presentations in the previous hour’ was the most reliable variable available for analysis.

**TABLE 1:** Characteristics of First Nations presentations to ED 2016–2020 (*n* = 6446)

	Waited, *n* (%)	Take own leave, *n* (%)	Unadjusted OR (95% CI)	*P*‐value	Adjusted OR (95% CI)	*P*‐value
All	5589 (87)	857 (13)				
Group						
Aboriginal	5226 (94)	799 (94)	1			
Aboriginal and Torres St Islander	315 (5)	47 (5)	1.05 (0.75–1.4)	0.88		
Torres St Islander	48 (1)	11 (1)	0.67 (0.35–1.29)	0.23		
Age						
0–39 years	2627 (47)	409 (48)	1		1	
40–59 years	2270 (41)	388 (45)	1.10 (0.95–1.28)	0.222	1.01 (0.86–1.19)	0.878
60+ years	692 (12)	60 (7)	0.56 (0.42–0.74)	<0.001	0.69 (0.51–0.93)	0.014
Sex						
Male	3195 (57)	463 (54)	1		1	
Female	2392 (43)	393 (46)	1.13 (0.98–1.31)	0.09	1.17 (1.01–1.36)	0.04
Marital status						
Not married	4682 (85)	727 (87)	1			
Married/*de facto*	804 (15)	113 (13)	0.91 (0.73–1.12)	0.357		
Health fund listed						
No	4958 (87)	806 (94)	1		1	
Yes	731 (13)	51 (6)	0.42 (0.31–0.56)	<0.001	0.61 (0.45–0.84)	<0.001
Next of kin available						
No			1		1	
Yes	4924 (88)	776 (91)	1.29 (1.02–1.65)	0.038	0.80 (0.61–1.05)	0.101
Safety alert						
No			1		1	
Yes	1144 (21)	282 (33)	1.91 (1.63–2.23)	<0.001	1.60 (1.35–1.90)	<0.001
Postcode						
Metropolitan Melbourne	4125 (74)	623 (74)	1			
Other	1464 (26)	234 (27)	0.95 (0.80–1.11)	0.492		
Usual accommodation						
Stable or private residential†	3921 (70)	464 (54)	1		1	
Homeless‡	781 (14)	199 (23)	2.15 (1.79–2.59)	<0.001	1.76 (1.44–2.15)	<0.001
Time of day						
06.00–18.00 hours	3352 (60)	398 (46)	1		1	
18.00–06.00 hours	2237 (40)	459 (54)	1.73 (1.50–2.00)	<0.001	1.67 (1.43–1.96)	<0.001
Source of attendance						
Self/other family friends	4165 (75)	760 (89)	1		1	
Police/Correctional	505 (9)	19 (2)	0.21 (0.13–0.33)	<0.001	0.43 (0.26–0.70)	<0.001
GP	335 (6)	27 (3)	0.44 (0.30–0.66)	<0.001	0.64 (0.42–0.97)	0.036
Other health professional	306 (6)	21 (3)	0.38 (0.24–0.59)	<0.001	0.51 (0.32–0.81)	0.004
Other§	278 (5)	30 (4)	0.59 (0.40–0.87)	0.007	0.64 (0.43–0.96)	0.029
Mode of arrival						
Ambulance and police	2490 (45)	245 (29)	1		1	
Public transport	647 (12)	119 (14)	1.8	NS	1.35 (1.04–1.75)	0.024
Other transport (includes walk‐ins)	944 (17)	212 (25)	2.82 (1.87–2.78)	<0.001	1.67 (1.34–2.08)	<0.001
Own transport	1508 (27)	282 (33)	1.89 (1.57–2.27)	<0.001	1.51 (1.23–1.86)	<0.001
Triage category						
1–3	3430 (61)	248 (29)	1		1	
4–5	2159 (39)	609 (71)	3.90 (3.33–4.57)	<0.001	3.17 (2.67–3.77)	<0.001

† Includes nursing home and prisons.

‡ Includes ‘public place’, ‘shelter/refuge’ and ‘boarding/hostel’.

§ For example, community services St Vincent's Hospital Melbourne staff.

CI, confidence interval; OR, odds ratio.

### Analysis

Counts and percentages are presented to describe the sample, with Pearson's chi‐squared and Fisher's exact test used to test association between categorical variables as appropriate. Crude and adjusted odds ratios (ORs) from logistic regressions were used to identify clinical and demographic predictors of a participant not completing care. Two‐tailed tests of significance at *P* < 0.05 were used in all inferential tests. All analyses were conducted using IBM SPSS Statistics version 27.0 (IBM, Armonk, NY, USA).

### Ethics approval

The St Vincent's Hospital Human Research Ethics Committee approved the project (HREC 041/20 No: 60925).

## Results

A total of 6446 presentations of First Nations patients occurred during the study period, constituting 3% of ED presentations. Of these, 5589 (87%) patients waited to be seen and 857 (13%) TOL (LNS, *n* = 624 [73%]; LOR, *n* = 233 [27%]).

Exploratory analysis comparing patients who LNS to those who LOR was undertaken. There were three differences. First, patients who LOR were more likely to do so during Wednesday to Friday and less likely to LOR on a Saturday/Sunday (LNS Wednesday–Friday, *n* = 225 [36%] *vs* LOR, *n* = 104 [45%], LNS Saturday/Sunday, *n* = 190 [30%] *vs* LOR, *n* = 49 [21%]). Second, LOR were more likely to happen 06.00–18.00 hours as compared to LNS (LNS, *n* = 267 [43%] *vs* LOR, *n* = 131 [56%]). Finally, LOR were more likely to be triage category 1–3 (LNS, *n* = 120 [19%] *vs* LOR, *n* = 128 [55%]). The two groups, patients who LNS and patients who LOR, were combined and considered as ‘TOL’ as all other demographic details were not significantly different.

Excluded from the analysis were patients who were assessed and received triage advice only (*n* = 58). At the study site, these patients are assessed by a senior ED nurse, often with medical input. ‘Triage advice only’ is allocated where it is decided that ED care is not required, and their needs have been met (e.g. a cut that requires a simple dressing and not a suture). If the patient expresses ongoing desire to see a doctor for further assessment, then they are registered for such. One patient who was an outlier presented 143 times during the study period and was also excluded.

A total of 6025 (94%) episodes involved patients who identified as Aboriginal, 362 (5%) were Aboriginal and Torres Strait Islander and 59 (1%) were Torres Strait Islander. These proportions of patients of Aboriginal or Torres Strait Islander origin were identical to those who waited and LNS (Table 1). Eight of the 6446 presentations required an Auslan interpreter, and all eight presentations waited to be seen. No other patients were recorded as requesting an interpreter. A total of 5409 (84%) of all presentations identified as not married or *de facto*.

Characteristics of the presentations of those who waited compared to patients who TOL are shown in Table 1. Multiple logistic regression analysis showed that patients who TOL were less likely to be over 60 years of age (adjusted OR 0.69, 95% confidence interval [CI] 0.51–0.93, *P* = 0.014), and slightly more likely to be women (adjusted OR 1.17, 95% CI 1.01–1.36, *P* = 0.04). Having private health insurance was predictive of having over a third less risk of leaving unseen (adjusted OR 0.60, 95% CI 0.45–0.84, *P* < 0.001). Having a safety alert was 1.6 times more likely to result in TOL. Of note, those patients triaged as categories 4 or 5 were significantly more likely to TOL (adjusted OR 3.17, 95% CI 2.67–3.77, *P* < 0.001).

Regarding ED ‘busyness’, there was no difference in the number of presentations in the hour prior to presentation for those who waited (median = 7, interquartile range 5–9) and those who did not (median = 7, interquartile range 5–10).

We examined the number of presentations per patient who waited as compared to those patients who TOL over the 5‐year period. A total of 35% of patients who TOL presented for that single episode. This was compared to only 23% of patients who waited and presented once to the ED in the 5‐year period (Table 2).

**TABLE 2:** Distributions of ED presentations over the study period (*n* = 6446)

	Waited	TOL
	*n* (%)	*n* (%)
Total	5589 (100)	857 (100)
1	1299 (23)	303 (35)
2	744 (13)	128 (15)
3	492 (9)	93 (11)
4	372 (7)	48 (6)
5	326 (6)	80 (9)
6–10	767 (14)	91 (10)
11–20	560 (10)	65 (8)
21–30	503 (9)	49 (6)
31–40	172 (3)	
>40	354 (6)	

TOL, take their own leave.

Presentations that TOL were more likely to represent within a 7‐day period (OR 2.46, 95% CI 2.08–3.01, *P* < 0.001). The most common time to represent was within day 0–1 (OR 2.63, 95% CI 2.3–3.15, *P* < 0.05). Of those representations that occur within 7 days, presentations that TOL are likely to result in a further TOL episode for the second presentation as compared with those that waited (OR 2.07, 95% CI 1.41–3.04, *P* < 0.01).

## Discussion

The present study is the first study to compare First Nations patients who TOL with those who completed treatment. It provides a comprehensive description of all leave events for the First Nations population of an inner‐city Australian ED over a 5‐year period. The rate of TOL for First Nations patients was higher (13%) than previously reported for Australian Metropolitan EDs (10%).^3^

### Characteristics

The sex characteristics in the present study do not replicate findings from other TOL Australian studies of the general population that show an increased prevalence of younger men leaving ED.^12^, ^13^, ^14^ Wright *et al*. found no difference between rate of Aboriginal men and women who leave unseen.^15^ Our findings show a small significant difference in that more female presentations TOL. This difference may not be clinically relevant. Mondays, Tuesdays and overnight were found to be the most common time periods that leave events occurred, consistent with studies of both the general and First Nations populations.^15^, ^16^ These times and days coincide with when the ED is at its busiest, supporting findings from previous studies whereby wait time is the biggest predictor of LNS.^17^ Additionally, patients assigned a triage category of 4 or 5 are at least three times as likely to TOL compared to those assigned category 1–3. Previous studies have found that race can be associated with a lower triage category.^4^, ^5^ Furthermore, in Australia, First Nations populations in inner‐city ED are more likely to be triaged to less urgent categories compared to those in remote areas.^2^ It is notable that patients who LOR were triaged at a more urgent level than those who LNS (LNS triage category 1–3 [19%] *vs* LOR triage category 1–3 [55%]). This suggests that even when patients are seen faster at less busy times, First Nations patients leave before treatment is completed. Interventions that solely address the LNS problem with increased accessibility without attempting any service change may increase the LOR rate.

### Social determinants of health

People who experience homelessness have the least resources to attend to their medical problems, reduced access to all healthcare services and a greater rate of TOL. This is a finding reflected in national data, with increasing rates of homeless patients among those who LNS.^2^ Additionally, in our study, it was found that having ‘Other’ housing status was also predictive of patients who TOL. This situation most commonly occurs when the patient is too sick, intoxicated or mentally unstable to communicate their housing status at the time of registration. The present study found that First Nations patients who do not have private health insurance are less likely to stay for treatment. Although this may indicate higher socio‐economic status in the general Australian population,^18^ this has not been established for First Nations patients.

### Behavioural alerts, miscommunication and unmet needs

We found patients with safety and security alerts were 1.6 times more likely to TOL (Table 1). Although alerts can contribute to a safer workplace for staff, implicit or unconscious bias may contribute to how patients with an alert are triaged and managed.^19^ In a recent international study of patients who TOL the most common reason for leaving ED after wait times (23%) was unmet expectations (22%) and negative interactions with ED staff (15%).^20^ Similar reasons are reiterated and amplified in First Nations populations in which reasons to leave include institutionalised racism, a lack of cultural safety, distrust of the health system, miscommunication and a lack of understanding of the treatment they were receiving.^21^ There is potential for both miscommunication and unmet needs occurring in these situations, with direct links to worse patient outcomes.

### Health service use

Our study population presented frequently to ED, with nearly half (46%) presenting more than six times over the 5‐year period. It was common for patients who TOL to present in the following days to the same ED (27%). Our study results are lower than other reports of 50–60% of LNS patients returning in the short term to the same ED.^12^, ^14^, ^22^ The most common time for return in our study was within 0–1 days for TOL presentations (Table 3). This replicates the time of return from other studies of emergency patients who LNS.^14^, ^22^, ^23^ It may be that in leaving ED, some of these patients have self‐identified that their need is best addressed in other healthcare settings, and indeed Lee *et al*. reported that one third of patients felt their problems were not suitable for ED.^22^ Previous studies have mixed findings regarding adverse outcomes,^24^ but it is uncommon for deaths and adverse events to occur in patients who TOL.^14^, ^25^ Our study illustrated patients who self‐referred and arrived by their own transport were more likely to leave unseen. This is contrary to Wright *et al*. who found patients from a rural Australian hospital who left unseen were more likely to have arrived by ambulance.^26^ This difference may reflect how ambulances may be used in rural *versus* metropolitan settings, coupled with the study ED proximity to public transport. We assumed return presentations in the short‐term were for the same condition, so that estimates of the admission rate may represent morbidity. In the current study, 9% of patients who TOL were subsequently admitted to a medical ward and 4% were admitted to a psychiatric ward. This is similar to a study by Gilligan *et al*. who reported that 5% of patients represented with mental health‐related conditions for admission.^27^ In our study 12% of patients required an admission to the ED short‐stay ward, suggesting a presentation that is less likely to be suitable for primary care.

**TABLE 3:** Representation within 7 days: bivariate regression analysis

	Original ED/admission status		OR (95% CI), *P*‐value
	Representations of those who waited, *n* (%)	Representation of those who TOL, *n* (%)	
Any representation <7 days	737 (13)	233 (27)	2.46 (2.08–3.01), *P* < 0.001
Days to representation <7 days			<0.001
0	101 (14)	58 (25)	1.00, *P* < 0.001
1	184 (25)	71 (31)	3.31 (1.58–7.01), *P* < 0.005
2	134 (18)	33 (14)	2.24 (1.08–4.62), *P* < 0.05
3	99 (13)	21 (9)	1.43 (0.66–3.09), NS
4	83 (11)	21 (9)	1.23 (0.54–2.79), NS
5	78 (11)	19 (8)	1.47 (0.64–3.35), NS
6	58 (8)	10 (4)	1.41 (0.61–3.27), NS
Discharge destination of representation, *n* (%)	*n* = 737	*n* = 233	
Home	338 (45.9)	103 (44.2)	NS
Left not seen	86 (11.7)	50 (21.5)	2.07 (1.41–3.04), *P* < 0.001
Left partial treatment	22 (3.0)	17 (7.3)	2.56 (1.33–4.90), *P* < 0.005
Triage advice only	18 (2.4)	4 (1.7)	NS
Short‐stay admission	143 (19.4)	28 (12.0)	0.57 (0.37–0.88), *P* < 0.05
Admit to medical ward	67 (9.1)	20 (8.6)	NS
Admit to psychiatric ward	18 (2.4)	10 (4.3)	NS
Residential care respite	0	1 (0.4)	NS
Return to prison/hostel/nursing home	31 (4.1)	0 (0.0)	0.04 (0.00–0.79), *P* < 0.05
ICU or operation theatre	6 (0.8)	0 (0.0)	NS
Return to ward	8 (1.1)	NA	NS

CI, confidence interval; NA, not available; NS, not significant; OR, odds ratio; TOL, take their own leave.

### Solutions and further research

The characteristics of First Nations patients who TOL in this inner‐city ED demonstrate a link to social and cultural determinants of health, and that the longer the wait times for being seen, the more likely it is a patient will TOL. In addition to patient‐centred, trauma‐informed care, we can commence the practice of triaging First Nations patients as a minimum at category 3. For our site this would represent a change of triage of approximately four patients every 2 days. This could be implemented at all hours of the day and is independent of Aboriginal Health Liaison service availability. Additional system changes that could encourage patients to stay include early engagement of the Aboriginal Health Liaison Officer service and other Aboriginal healthcare workers to overcome miscommunication, undertaking advocacy aligned with cultural protocols and providing a more welcoming environment for patients.^28^, ^29^ Innovative programmes such as the ‘Flexiclinic’ reduce length of wait, miscommunication and unmet needs.^5^, ^6^ Further clinical research is required to assess whether making a minimum admission triage category 3 and creating a more welcoming environment can encourage Aboriginal and Torres Strait Islander Victorians to stay and receive full health benefits from their engagement with ED.^29^

### Strengths and limitations

Limitations of the study include that the clinical data were not collected solely for the purpose of the study. The waiting time entered for the LNS patients did not always reflect the participants true departure time. This has resulted in the exclusion of a ‘length of wait time’ variable. However, ‘length of wait time’ is universally accepted to be one of the most important factors associated with patients who leave unseen from the ED.^14^, ^16^, ^17^, ^20^, ^24^ We used ‘Presentations in the previous hour’ as a marker of ‘ED Busyness’ because availability, but this measure has well‐documented limitations.^13^, ^30^ More robust ‘busyness’ markers such as ‘ED occupancy’ at the time of triage for each patient would have been preferable.^30^ Potential bias may have occurred as the demographic data is relevant only at time of data retrieval and may have changed over the 5‐year study period to when the patient presented. Furthermore, the study was conducted at a single inner‐city hospital. Finally, it is possible that patients who are Aboriginal or Torres Strait Islander were not asked about their identity, or chose not to disclose it, when attending the ED. Their experience was not included in this work. A strength of the present study is the size of the sample and the lengthy study period of 5 years. We deliberately used strengths‐based narratives to frame our methodological approach and study structure.^8^

## Conclusion

This is the first study to attempt to characterise why some First Nations patients complete their care in the ED while others do not. Future qualitative research is required to explore the ED experience for First Nations patients; encompassing arrival, triage, waiting and care delivery. This will shed light on many more factors contributing to why some patients prematurely leave and why some stay. We recommend a strengths‐based framing for all research about First Nations health inequities in emergency care, so that findings can inform positive actions to improve care experience and outcomes for all Aboriginal and Torres Strait Islander people.

## Data Availability

Author elects not to share data.
